# Gastroenteritis Outbreaks in 2 Tourist Resorts, Dominican Republic

**DOI:** 10.3201/eid1511.090350

**Published:** 2009-11

**Authors:** Antonio Doménech-Sánchez, Carlos Juan, Antonio J. Rullán, José L. Pérez, Clara I. Berrocal

**Affiliations:** Saniconsult Ibérica SL, Palma de Mallorca, Spain (A. Doménech-Sánchez, C.I. Berrocal); Instituto Universitario de Investigación en Ciencias de la Salud, Palma de Mallorca (A. Doménech-Sánchez, C. Juan, J.L. Pérez); Hospital Son Dureta. Palma de Mallorca (C. Juan, J.L. Pérez); Servicio Navarro de Salud, Pamplona, Spain (A.J. Rullán)

**Keywords:** norovirus outbreak, gastrointestinal illness, molecular methods, tourist resort, Dominican Republic, viruses, letter

**To the Editor:** Noroviruses are an important cause of acute gastroenteritis, and outbreaks caused by these viruses have emerged as a major challenge to the healthcare, leisure, and tourism industries. The primary reason is their highly efficient transmission among persons in semiclosed populations such as those in healthcare facilities, hotels, and cruise ships. During an outbreak, primary cases result from exposure to a fecally contaminated vehicle (e.g., food or water), whereas secondary and tertiary cases among contacts of primary case-patients result from person-to-person transmission (*1*). Airborne and fomite transmission also play a role in the virus spread during outbreaks (*2*). Transmission through recreational water has also been described (*3*).

We investigated 2 outbreaks of norovirus gastroenteritis in tourist resorts in the Dominican Republic in January 2005. A total of 402 persons and 371 persons at 2 resorts, 1 located in Punta Cana (attack rate 6.8%) and another in Puerto Plata (attack rate 6.2%), respectively, reported symptoms of diarrhea, vomiting, headache, and fatigue. A total of 35 stools samples, 28 from Punta Cana and 7 from Puerto Plata, were negative for bacterial or parasitic pathogens. However, norovirus was confirmed by the IDEIA norovirus immunoassay (DakoCytomation, Ely, UK) in 11 samples from Punta Cana and 7 samples from Puerto Plata.

Active measures to reduce norovirus transmission were adopted by the 2 resorts, including an increase in cleaning frequency and increase in concentration of chlorine used for surface disinfection of public areas (1,000 mg/L), kitchenware (200 mg/L for 15 min), and fruits and vegetables (150 mg/L for 15 min). Personnel involved in food preparation were invited to fill out an epidemiologic questionnaire and provide stool samples.

Because these preventive measures were not effective, water was suspected as a possible route of transmission, and diverse approaches were used to isolate viral RNA from different types of water. For sewage water, 100 mL were concentrated by a Molecular Weight Cutoff (MWCO) 100,000 Da Vivaspin20 centrifugal concentrator (Sartorius, Madrid, Spain) to a 5-mL concentrated sample. Five milliliters of chloroform:isoamyl alcohol (24:1) was added to the sample, mixed in a vortex, and centrifuged at 1,000 × *g* for 10 min (*4*). A second concentration step to 1 mL was performed with the resulting aqueous phase using a MWCO 100,000 Da Vivaspin6 centrifugal concentrator (Sartorius). For tap and recreational water, a sample of 2 L was processed through a cellulose nitrate filter, as described (*5*). The filter was first activated by 5 mL of 0.25 mol/L AlCl_3_. After a washing step with 200 mL 0.5 mmol/L H_2_SO_4_, viruses were eluted from the filter with 10 mL of 1 mmol/L NaOH and neutralized with 50 µL of 0.1 mol/L H_2_SO_4_ and 1.1 mL 10× pH 8.0 Tris-EDTA buffer. Finally, samples were concentrated to a final volume of 1 mL with a Vivaspin6, centrifugal concentrator, as described above.

Viral RNA was isolated by the QIAamp Viral RNA Mini Kit (QIAGEN, Hilden, Germany), treated with Dnase I (Invitrogen, Carlsbad, CA, USA), and reverse transcribed to cDNA with Superscript II reverse transcriptase (Invitrogen). Finally, cDNA was amplified by multiplex-PCR with Taq Gold polymerase (Applied Biosystems, Foster City, CA, USA) by using forward primers Nor31N (5′-CAGATTAYACWGCWTGGGA-3′) and Nor32M (5′-CAGATTAYTCWCGWTGGGA-3′); and reverse primers Nor41M (5′-CCARTGATTTATGCTGTTCAC-3′) and Nor42N (5′-CCAGTGGGCGATGGAGTTC-3′), specific for the RNA polymerase gene (provided by J.A. Boga and M. Oña) (*6*). PCR was performed with the following conditions: 12 min at 94°C, (1 min at 94°C, 1 min at 55°C, 1 min at 72°C) for 40 cycles, and 10 min at 72°C. PCR products were analyzed by agarose gel electrophoresis. A band of 221 bp was considered a positive result for norovirus.

Norovirus was detected in the 4 samples of sewage water analyzed (2 from each location) collected after intervention, indicating that viral carriers still remained in the resorts. Moreover, norovirus particles were detected in the 2 depurated water samples (1 from each area), then indicating insufficient treatment conditions. Further investigation showed that depurated water was being used to water plants and grass by sprinkling, and becoming an important secondary source for infection. Norovirus-contaminated sewage was treated with different concentrations of chlorine and reverse transcription–PCR demonstrated that 15 mg/L for 1 h did not give a positive signal. Garden sprinkling watering was replaced by inundation watering. Although the virus was not detected either in tap or recreational water, hyperchlorination was carried out to prevent possible dissemination of norovirus. Preventive measures described above for surfaces, kitchenware and food were maintained for 2 additional weeks, and no additional cases were detected after 1 month had passed since the first case. To summarize, only interventions at multiple points, including eradication of secondary sources, and preventive measures to avoid person-to-person transmission enabled the outbreaks to be controlled.
